# 2315: Documenting ADAPT (Addressing Disparities in Asian Populations through Translational research): The growth of a community-research collaborative

**DOI:** 10.1017/cts.2017.172

**Published:** 2018-05-10

**Authors:** Amy LeClair, Carolyn Rubin

**Affiliations:** Tufts University, Medford, MA, USA

## Abstract

OBJECTIVES/SPECIFIC AIMS: Addressing Disparities in Asian Populations through Translation research (ADAPT) is a community-research partnership funded by the Tufts Clinical Translational Sciences Institute (CTSI). Founded in 2011, this collaborative brings together 7 Chinatown-serving community-based organizations and academic researchers with the goal of improving health for the local Chinatown community and beyond. The goal of this research project was to document the best practices, lessons learned, and process through which ADAPT has developed and grown. The aim of this project is to disseminate the model to other CTSAs who are currently engaged in METHODS/STUDY POPULATION: We used a combination of qualitative interviews and content analysis to gather data on the evolution of ADAPT over the last 5 years. Current members from both community organizations and the university/medical center were interviewed about their experiences participating in ADAPT. When possible, interviews were recorded and transcribed verbatim. Deidentified transcripts and administrative documents including meeting minutes, conference summaries, bylaws, and mission statements were coded using Dedoose analytic software. RESULTS/ANTICIPATED RESULTS: Established community-based participatory research (CBPR) principles, including mutual respect, transparency, and commitment, are viewed as necessary, but not sufficient. Patience—both with other members and with the group as a work in progress—is highlighted as being a necessary characteristic of participants. Time and funding are 2 of the most important resources, and the majority of members agree that there is no substitute for “skin in the game.” Attempts at last minute, opportunistic engagement were provided as examples of what had not worked. One ongoing tension is the balance between process and product. Individual members are beholden to organizations to different degrees, and the need to produce something in the form of publications or grant money can limit the amount of time members can commit to the collaborative. At the same time, these products are unlikely to materialize if members are not invested in the process of growing and sustaining the collective. DISCUSSION/SIGNIFICANCE OF IMPACT: Out of the 7 community organizations who currently participate in ADAPT, only 1 is explicitly focused on health in the traditional sense. The others are primary service organizations, but because they understand the impact of the social determinants of health on the local community—including housing, employment, education, nutrition, among other factors—the research collaborative is able to leverage the knowledge and expertise of the academic researchers and the community partners to focus on health topics most salient to the local Chinatown community.

